# Supplementary material for: RNF213 promotes Treg cell differentiation by facilitating K63-linked ubiquitination and nuclear translocation of FOXO1
Source: Nat Commun. 2024 Jul 16;15:5961. doi: 10.1038/s41467-024-50392-z (PMC11252262; doi:10.1038/s41467-024-50392-z)
Supplement: Supplementary file 1 — Supplementary Information [file 41467_2024_50392_MOESM1_ESM.pdf]

Supplemental information

RNF213 promotes Treg cell differentiation by facilitating nuclear translocation of FOXO1 through K63-linked ubiquitination

Xiaofang Yang[1#], Xiaotong Zhu[2#], Junli Sheng[1#], Yuling Fu[1], Dingnai Nie[1], Xiaolong You[1], Yitian Chen[1], Xiaodan Yang[1], Qiao Ling[1], Huili Zhang[3]*, Xiaomin Li[4]*, Shengfeng Hu[1, 2]*

Supplementary figure 1

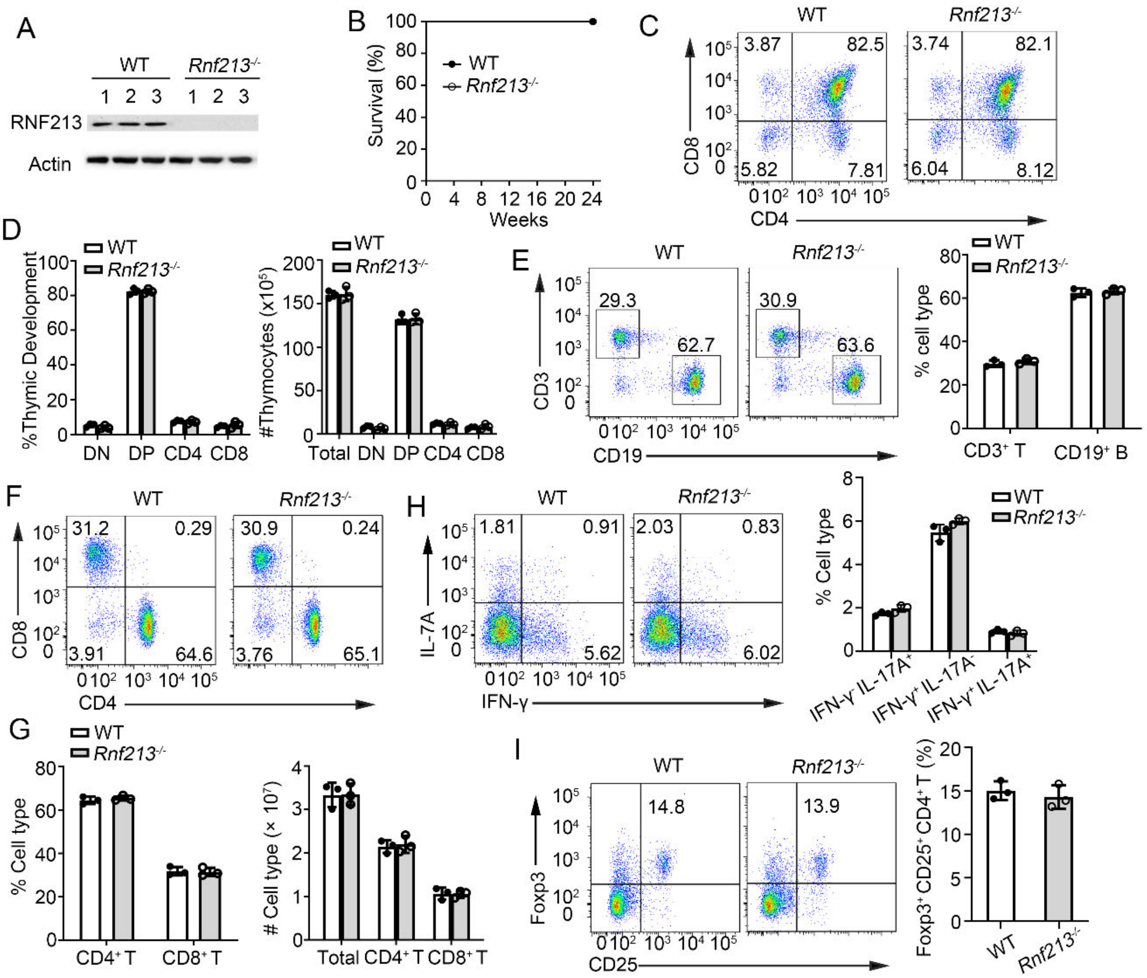

Supplementary Figure 1, Related to Figure 1. RNF213-deficiency did not affect normal T cell homeostasis. (A) RNF213 immunoblot using $CD3^+$ T cells or $CD4^+$ cells from spleens from naive WT and *Rnf213*$^{-/-}$ mice. Lane represents an individual mouse. **(B)** Survival curve of WT and *Rnf213*$^{-/-}$ mice. **(C)** Representative expression of CD4 and CD8 from thymocytes of WT and *Rnf213*$^{-/-}$ mice (3 weeks old). **(D)** Percentages (left) and total numbers (right) of thymocytes in each stage of thymic development (n = 3 mice per genotype). DN, double negative ($CD4^-$ $CD8^-$); DP, double positive ($CD4^+$ $CD8^+$); CD4, CD4 single positive ($CD4^+$ $CD8^-$); CD8, CD8 single positive ($CD4^-$ $CD8^+$). **(E)** Representative expression of $CD3^+$ T and $CD19^+$ B cells from splenocytes of WT and *Rnf213*$^{-/-}$ mice (8-10 weeks old). **(F-G)** Representative expression of $CD4^+$ T and $CD8^+$ T (Gated in $CD3^+$ T cells) from splenocytes of WT and *Rnf213*$^{-/-}$ mice (8-10 weeks old) **(F)**. Pooled data of

percentages and total numbers are presented in **(G)**. **(H)** Cells from the spleens were brief stimulated with PMA/ionomycin ex vivo and the intracellular production of IFN-γ and IL-17A by $CD4^+$ T cells was determined. Pooled data are presented in the right panel. **(I)** Expression of CD25 and Foxp3 were detected on $CD4^+$ T cells from spleens. Pooled data are presented in the below panel. Data shown are the mean ±SD. Data are representative of three independent experiments with similar results. Source data are provided as a Source Data file.

Supplementary figure 2

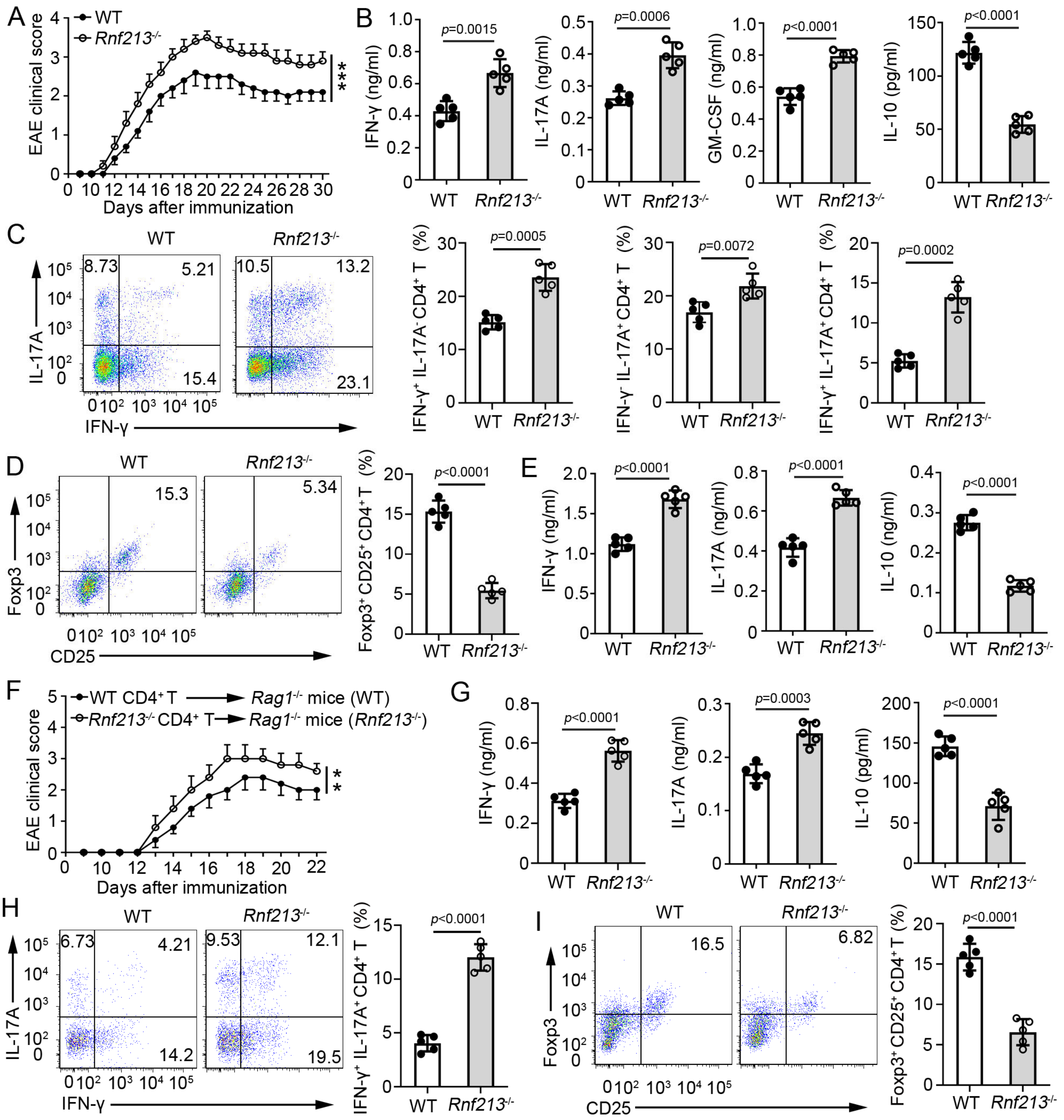

Supplementary Figure 2, Related to Figure 1. RNF213-deficient promoted EAE development through regulating Treg cell differentiation. (A-E) WT and *Rnf213*$^{-/-}$ mice were immunized with MOG(35-55) peptide in CFA adjuvant and pertussis toxin to induce EAE. **(A)** The graph shows the clinical score of EAE (n = 5 per group). **(B)** Concentration of IFN-γ, IL-17, GM-CSF and IL-10 in serum was measured by ELISA on day 20. **(C)** The cells infiltrating to central nervous system (CNS) were restimulated with MOG(35-55) peptide directly ex vivo, and the intracellular production of IFN-γ and IL-17A by CD4^{+} T cells was determined. Pooled data are

presented in the right panel. **(D)** Flow cytometry of Treg cell ($CD25^+$ $Foxp3^+$) in CNS of WT and *Rnf213*$^{-/-}$ mice on days 20 during EAE. Pooled data are presented in the right panel. **(E)** The cells infiltrating to CNS were stimulated for 48 hours with MOG(35-55) peptide and cytokine productions were measured by ELISA. **(F-I)** Purified WT or *Rnf213*$^{-/-}$ naïve $CD4^+$ T cells were adoptively transferred into *Rag1*$^{-/-}$ mice. Recipient mice were immunized with MOG(35-55) peptide in CFA adjuvant and pertussis toxin to induce EAE. **(F)** The graph shows the clinical score of EAE (n = 5 per group). **(G)** Concentration of IFN-γ, IL-17, GM-CSF and IL-10 in serum was measured by ELISA on day 20. **(H-I)** Flow cytometry of $IFN\gamma^+$, $IL\text{-}17A^+$ $CD4^+$ cells **(H)** or $CD4^+$ $CD25^+$ $Foxp3^+$ Treg cells **(I)** in CNS of WT and *Rnf213*$^{-/-}$ mice on days 20 during EAE. Pooled data are presented in the right panel. Data shown are the mean ±SD. $^*P < 0.05$, $^{**}P < 0.01$ and $^{***}P < 0.001$. *P* values were calculated using two-side, unpaired Student's *t*-test **(A-I)**. N = 5 **(A-I)** repeats from three independent experiments. Source data are provided as a Source Data file.

Supplementary figure 3

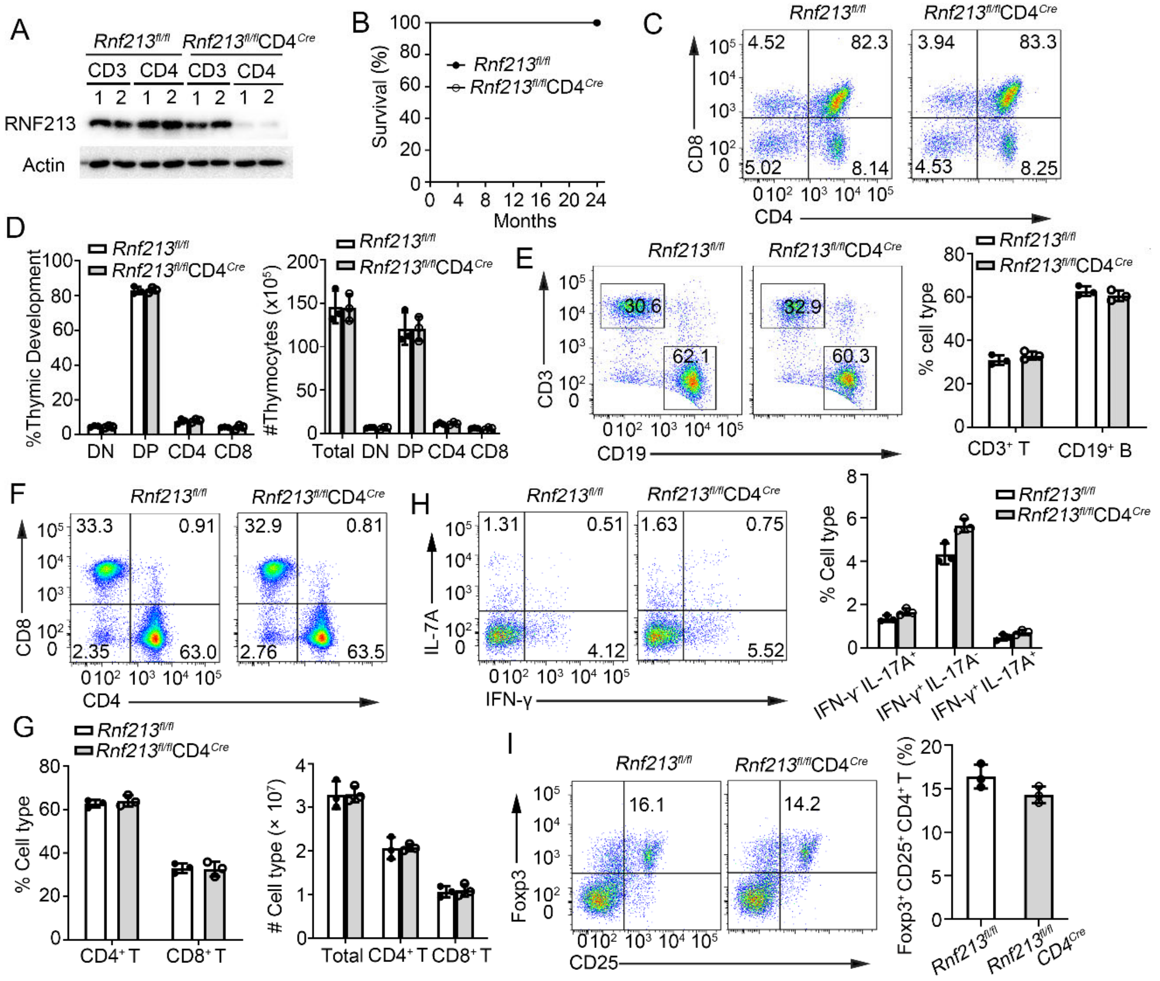

Supplementary Figure 3, Related to Figure 1. RNF213-deficiency in CD4$^+$ T did not affect normal T cell homeostasis. (A) RNF213 immunoblot using CD3$^+$ T cells or CD4$^+$ cells from spleens from naive *Rnf213$^{fl/fl}$* and *Rnf213$^{fl/fl}$CD4^{Cre}* mice. Lane represents an individual mouse. **(B)** Survival curve of *Rnf213$^{fl/fl}$* and *Rnf213$^{fl/fl}$CD4^{Cre}* mice. **(C)** Representative expression of CD4 and CD8 from thymocytes of *Rnf213$^{fl/fl}$* and *Rnf213$^{fl/fl}$CD4^{Cre}* mice (3 weeks old). **(D)** Percentages (left) and total numbers (right) of thymocytes in each stage of thymic development (n = 3 mice per genotype). DN, double negative (CD4$^-$ CD8$^-$); DP, double positive (CD4$^+$ CD8$^+$); CD4, CD4 single positive (CD4$^+$ CD8$^-$); CD8, CD8 single positive (CD4$^-$ CD8$^+$). **(E)** Representative expression of CD3$^+$ T and CD19$^+$ B cells from splenocytes of *Rnf213$^{fl/fl}$* and *Rnf213$^{fl/fl}$CD4^{Cre}* mice (8-10 weeks old). **(F-G)** Representative expression of CD4$^+$ T and CD8$^+$ T (Gated in CD3$^+$ T cells) from splenocytes of

$Rnf213^{fl/fl}$ and $Rnf213^{fl/fl}CD4^{Cre}$ mice (8-10 weeks old) **(F)**. Pooled data of percentages and total numbers are presented in **(G)**. **(H)** Cells from the spleens were brief stimulated with PMA/ionomycin ex vivo and the intracellular production of IFN-γ and IL-17A by CD4^{+} T cells was determined. Pooled data are presented in the right panel. **(I)** Expression of CD25 and Foxp3 were detected on CD4^{+} T cells from spleens. Pooled data are presented in the below panel. Data shown are the mean ±SD. Data are representative of three independent experiments with similar results. Source data are provided as a Source Data file.

Supplementary figure 4

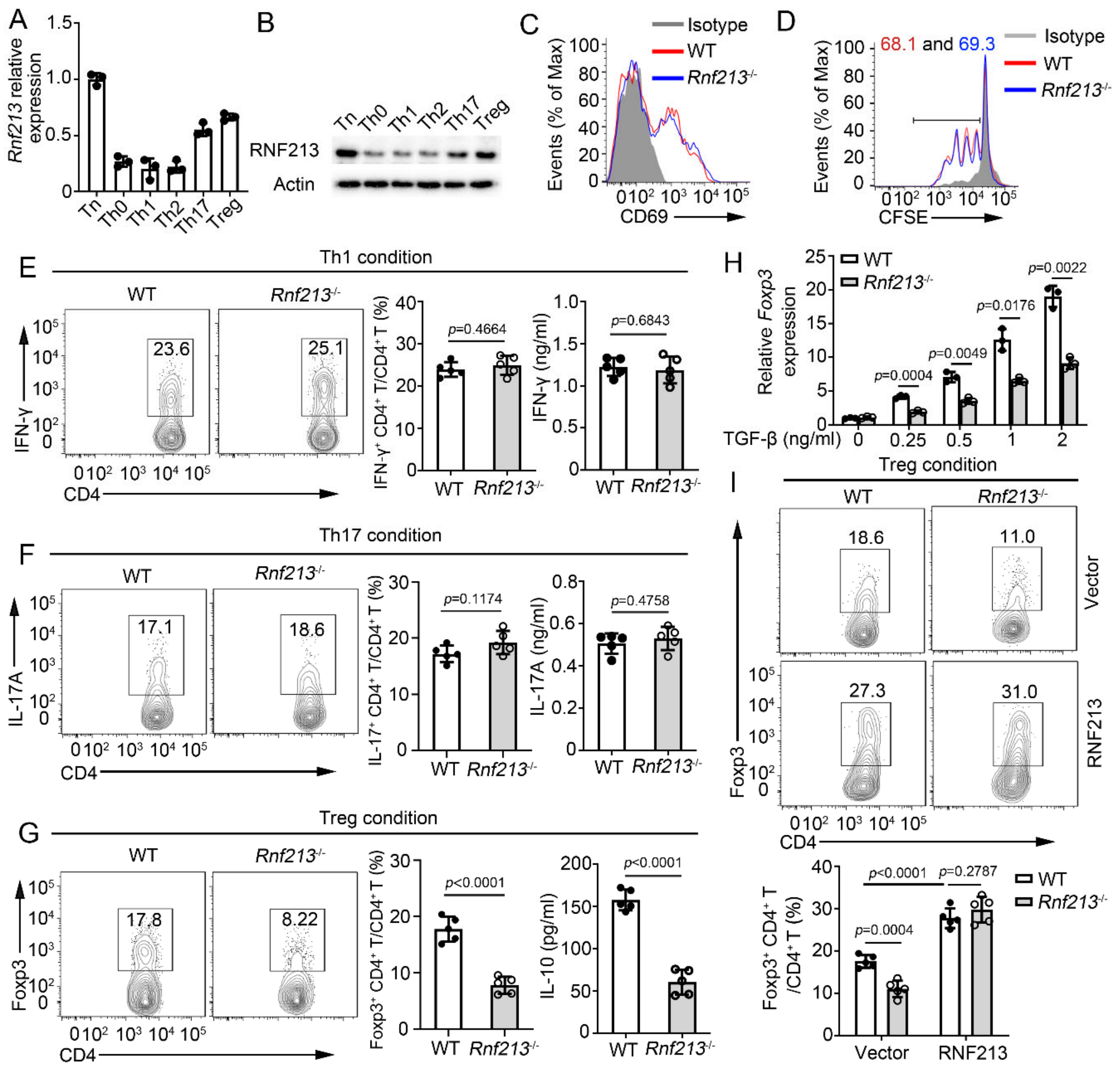

Supplementary Figure 4, Related to Figure 2. RNF213 deficiency in $CD4^+$ T cells decreased Treg cell differentiation *in vitro*. (A-B) Purified naïve $CD4^+$ T cells from WT mice were stimulated under standard Th0, Th1, Th2, Th17 or Treg conditions and harvested on day 5. RNF213 expression levels were detected by qPCR **(A)** or western blot **(B)**. **(C-D)** Purified naïve $CD4^+$ T cells isolated from WT or *Rnf213*$^{-/-}$ mice were stimulated with plate bound anti-CD3/CD28 for 72 hours. **(C)** Expression of activation marker CD69 by $CD4^+$ T cells were determined. **(D)** Isolated purified naïve $CD4^+$ T cells were labeled with CFSE, stimulated and determined by flow cytometry. **(E-G)** Purified naïve $CD4^+$ T cells from WT or *Rnf213*$^{-/-}$ mice were isolated, and stimulated under standard Th1, Th17 or Treg conditions and harvested on day 5. Flow

cytometry of intracellular IFN-γ **(E)**, IL-17A **(F)** or Foxp3 **(G)** and pooled data in $CD4^+$ T cells. **(H)** QPCR analysis of Foxp3 in WT and *Rnf213*$^{-/-}$ $CD4^+$ T cells activated with the indicated amounts of TGF-β for 5 days. **(I)** Flow cytometry of intracellular Foxp3 in WT or *Rnf213*$^{-/-}$ naive $CD4^+$ T cells infected with control retrovirus (Vector) or retrovirus expressing RNF213 and differentiated under standard Treg conditions. Pooled data are presented in the below panel. Data shown are the mean ±SD. $^*P < 0.05$, $^{**}P < 0.01$ and $^{***}P < 0.001$. *P* values were calculated using two-side, unpaired Student's *t*-test **(E-I)**. N = 3 **(A and H), n=5 (E-G, and I)** repeats from three independent experiments. Source data are provided as a Source Data file.

Supplementary figure 5

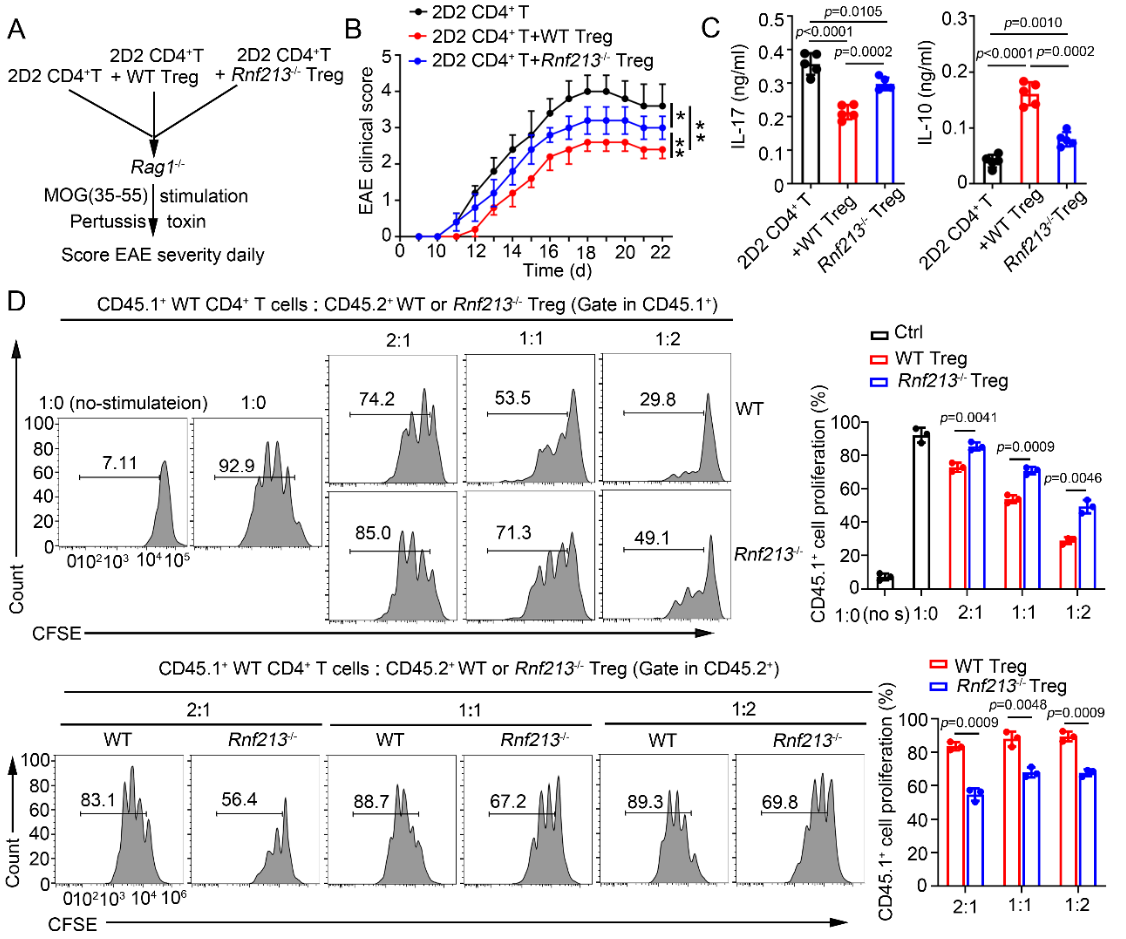

Supplementary figure 5, Related to Figure 3. RNF213 is essential for Treg cells to suppress T cell-mediated EAE. (A) Schema of EAE induction in *Rag1*$^{-/-}$ mice transferred with 2D2 $CD4^+$ T cells either alone or in combination with WT or *Rnf213*$^{-/-}$ $CD4^+$ $CD25^+$ Treg cells. **(B)** The graph shows the clinical score of EAE (n = 5 respectively). **(C)** Mice were harvested on day 28, and concentration of IL-17 and IL-10 in serum was measured by ELISA. **(D)** WT CD45.1$^+$ $CD4^+$ T cells were sorted, activated by CD3 and CD28 antibodies, and co-cultured with Treg cells that were sorted from WT or *Rnf213*$^{-/-}$ mice in an indicated ratio. The proliferation of $CD4^+$ T cells was assessed using CFSE dilution assay. And the percentages of proliferation of $CD4^+$ T were analysed. Data shown are the mean ±SD. *$P < 0.05$, **$P < 0.01$ and ***$P < 0.001$. P values were calculated using two-side, unpaired Student's *t*-test in **(B-D)**. N = 5 **(B-C)**, n = 3 **(D)** repeats from three independent experiments. Source data are provided as a Source Data file.

Supplementary figure 6

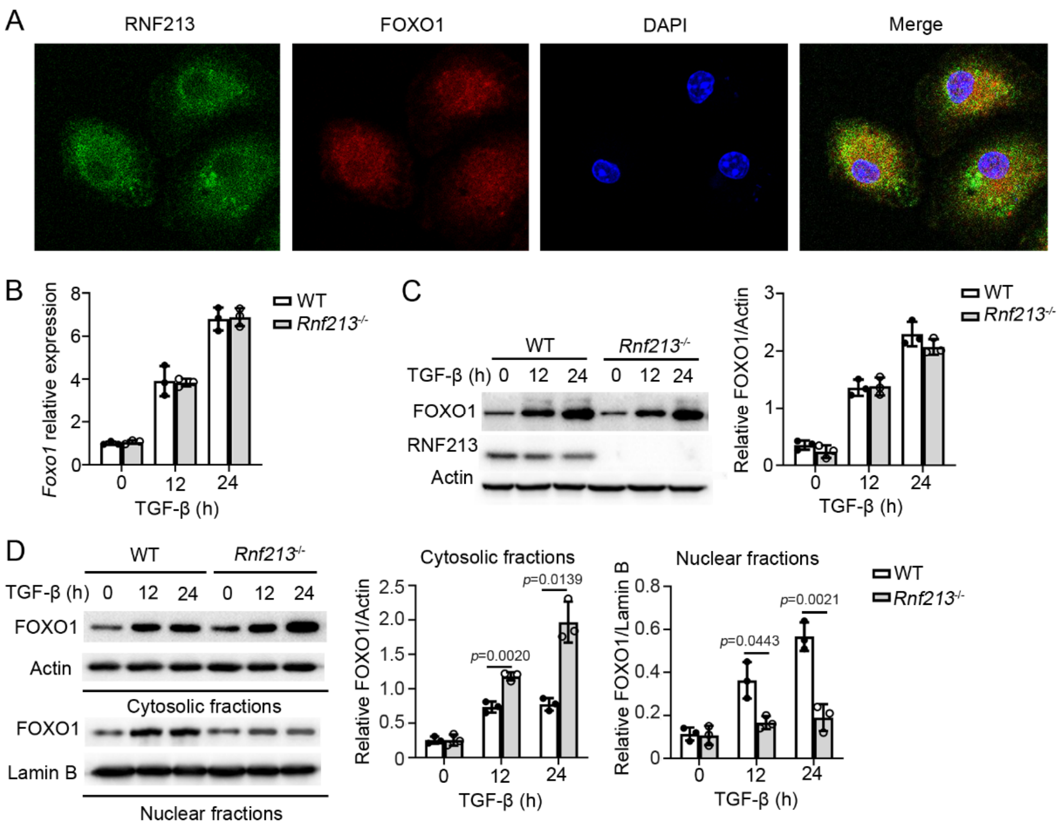

Supplementary figure 6, Related to Figure 4. RNF213 interacts with FOXO1 and is critical to regulate translocation of FOXO1 to the nucleus. (A) HEK293 cells transfected with the indicated plasmids were stained with anti-RNF213 (Green) Abs, anti-FOXO1 (red) Abs, and DAPI (nucleus, blue) and then observed by fluorescence microscopy. **(B-C) (B)** QPCR and Western blot **(C)** analysis of FOXO1 expression in CD4$^+$ cells from WT or *Rnf213*$^{-/-}$ mice differentiated under Treg-polarizing conditions for indicated times. **(C)** Luciferase activity of HEK293T cells transfected with a luciferase reporter driven by the Foxp3 promoter and expressing vector alone or various combinations (horizontal axis) of FOXO1 and RNF213. **(D)** Treg cells were double-stained with anti-FOXO1 (red) Abs and DAPI (nucleus, blue) and then observed by fluorescence microscopy. Scale bars: 50μm. Quantification of fluorescent digital imaging analysis of FOXO1 expression in the cytosol and the nucleus depicted as the percentage expression levels are presented in the right panel. Data shown are the mean ±SD. *$P < 0.05$, **$P < 0.01$ and ***$P < 0.001$. *P* values were calculated using two-side, unpaired Student's *t*-test in **(B-D)**. N = 3 **(B-D)** repeats from three independent experiments. Source data are provided as a Source Data file.

Supplementary figure 7

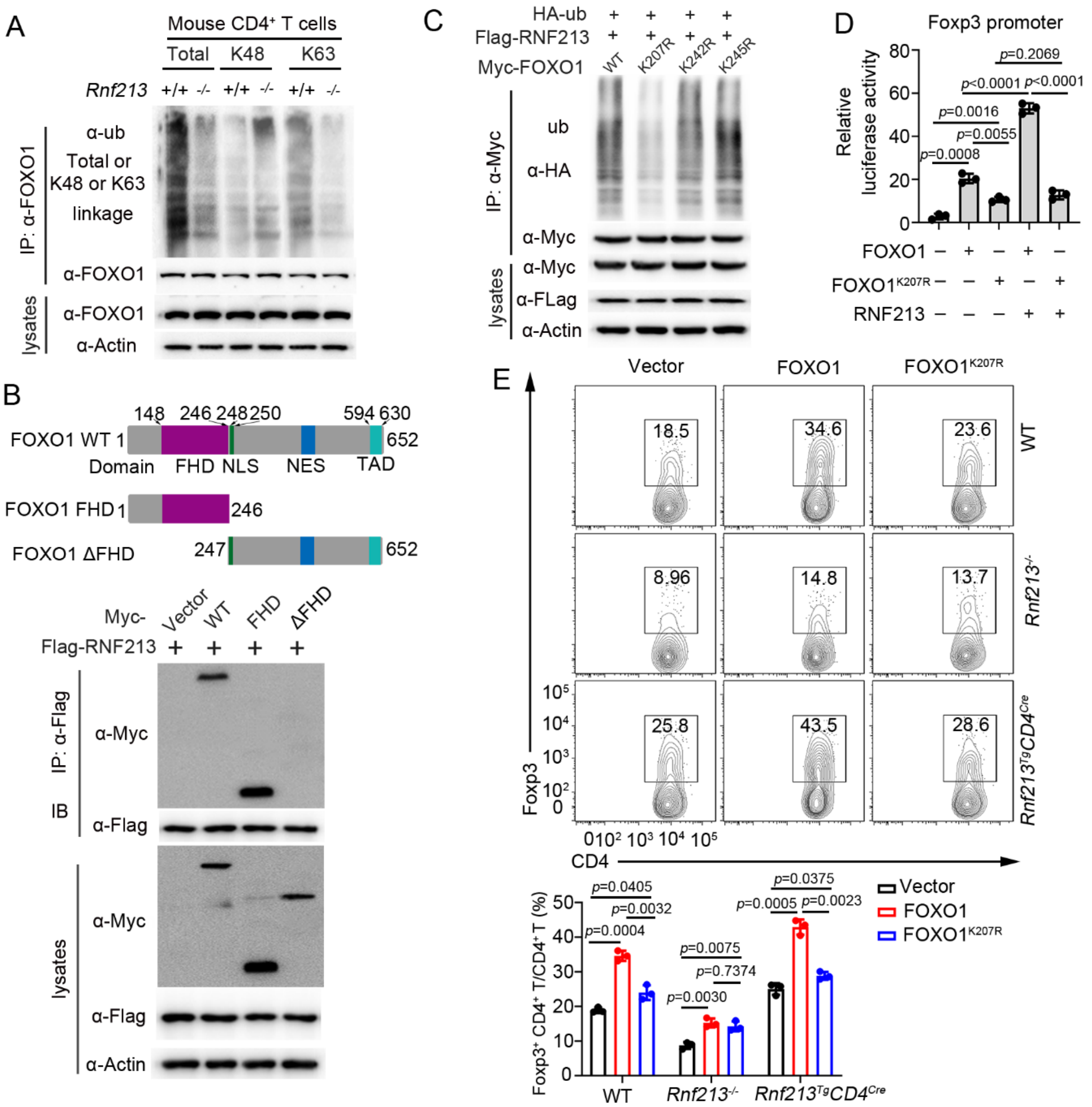

Supplementary figure 7, Related to Figure 6. RNF213 regulated Treg cell differentiation by regulating FOXO1 K63-linked polyubiquitination. (A) Denature- IP and IB analysis (with anti-K48 or K63 linkage polyubiquitin) of WT or *Rnf213*$^{-/-}$ CD4^{+} T cells stimulated with TGF-β plus anti-CD3/CD28 for 3 h. MG132 and BafA1 was added to inhabit ubiquitin-proteasome and autolysosome. **(B)** Immunoprecipitation (IP) and immunoblot (IB) analysis of HEK293T cells that were transfected with indicated plasmids for 24 hours. FHD, Forkhead (FH) domain; TAD: Transactivation domain. **(C)** Denature-IP and IB analysis of HEK 293T cells transfected with Myc-FOXO1 (WT) or mutations (K207R, K242R, K245R), HA-

ubiquitin (ub), and Flag-RNF213. MG132 and BafA1 was added to inhabit ubiquitin-proteasome and autolysosome. **(D)** Luciferase activity of HEK293T cells transfected with a luciferase reporter driven by the Foxp3 promoter and expressing vector alone or RNF213, FOXO1 and $FOXO1^{K207R}$. **(E)** Flow cytometry of Foxp3 in $CD4^+$ T cells infected with control retrovirus (Vector) or retrovirus expressing FOXO1 and $FOXO1^{K207R}$ differentiated under Treg-polarizing conditions. Pooled data are presented in the right panel. Data shown are the mean ±SD. *$P < 0.05$, **$P < 0.01$ and ***$P < 0.001$. *P* values were calculated using two-side, unpaired Student's *t*-test in **(D-E)**. N = 3 **(D-E)** repeats from three independent experiments. Source data are provided as a Source Data file.

Supplementary figure 8

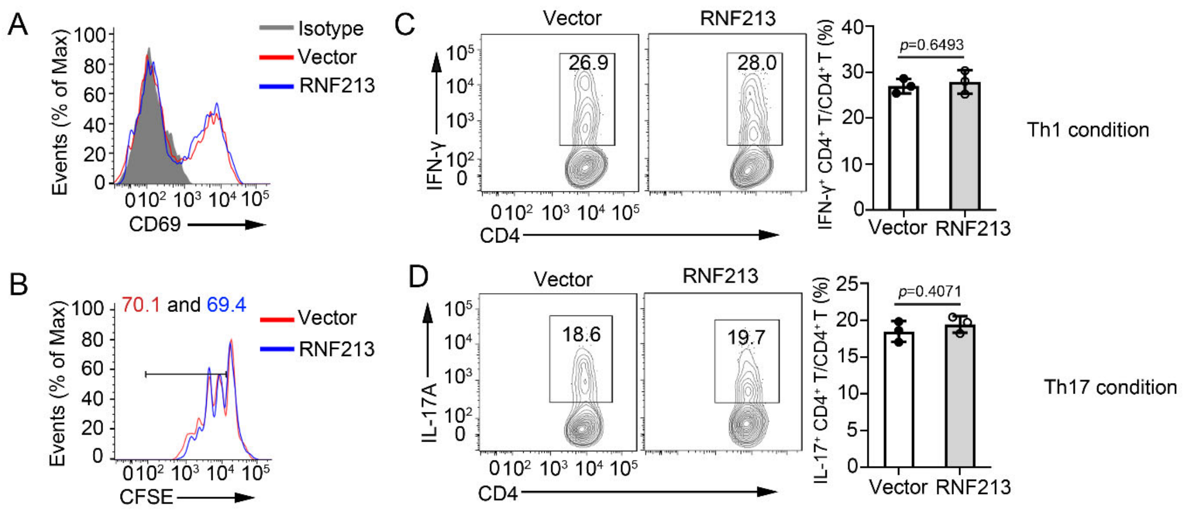

Supplementary figure 8, Related to Figure 7. RNF213 promoted Treg cell differentiation in human Tregs. (A-B) These CD4$^+$ T cells were stimulated with plate bound anti-CD3/CD28 for 72 hours. **(A)** Expression of activation marker CD69 by CD4$^+$ T cells were determined. **(B)** Isolated purified naïve CD4$^+$ T cells were labeled with CFSE, stimulated and determined by flow cytometry. **(C-D)** These CD4$^+$ T cells were stimulated under standard Th1 or Th17 conditions and harvested on day 5. Flow cytometry of intracellular IFN-γ **(C)** or IL-17A **(D)** and pooled data in CD4$^+$ T cells in the right panels. *$P < 0.05$, **$P < 0.01$ and ***$P < 0.001$. P values were calculated using two-side, unpaired Student's *t*-test in **(C-D)**. N = 3 **(C-D)** repeats from three independent experiments. Source data are provided as a Source Data file.

Supplementary figure 9

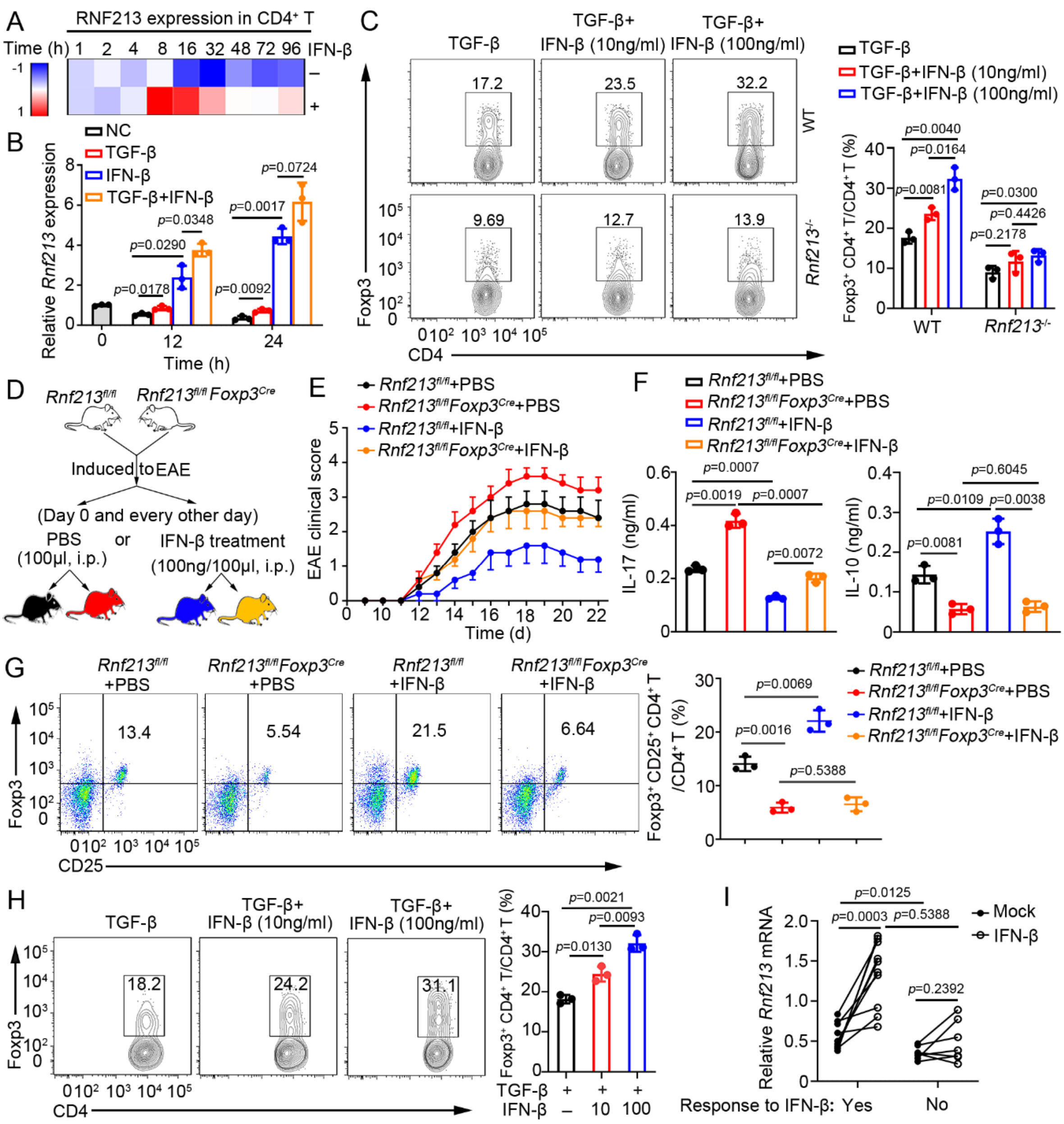

Supplementary figure 9. IFN-β promoted Treg cell differentiation by inducing RNF213 expression. (A) The heatmap displaying RNF213 expression is based on RNA-seq data comparing $CD4^+$ T cells stimulated with or without IFN-β (GSE195541). **(B)** QPCR analysis of RNF213 expression in $CD4^+$ cells differentiated under Treg-polarizing conditions without or with IFN-β (100ng/ml). **(C)** Flow cytometry of intracellular Foxp3 in $CD4^+$ cells differentiated under Treg-polarizing conditions without or with IFN-β (10 ng/ml or 100 ng/ml). Pooled data are presented in the right panel. **(D)** Schematic of experimental design for assessing the effects of

IFN-β treatment on EAE. **(E)** The graph shows the clinical score of EAE (n = 5 per group). **(F)** Concentration of IL-17 and IL-10 in serum was measured by ELISA on day 20. **(G)** Flow cytometry of Treg cell ($CD25^+$ $Foxp3^+$) in CNS on days 20 during EAE. Pooled data are presented in the right panel. **(H)** Flow cytometry of intracellular Foxp3 in human $CD4^+$ cells differentiated under Treg-polarizing conditions without or with IFN-β (10 ng/ml or 100 ng/ml). Pooled data are presented in the right panel. **(I)** QPCR analysis of RNF213 in human $CD4^+$ cells from patients with multiple sclerosis that responded (n=10) or did not respond (n=7) to IFN-β (100ng/ml). Data shown are the mean ±SD. *$P < 0.05$, **$P < 0.01$ and ***$P < 0.001$. *P* values were calculated using two-side, unpaired Student's *t*-test in **(B-H)**, and was calculated using paired Student's *t*-test in **(I)**. N = 3 **(B-C and F-H)** repeats from three independent experiments. Source data are provided as a Source Data file.

Supplementary figure 10

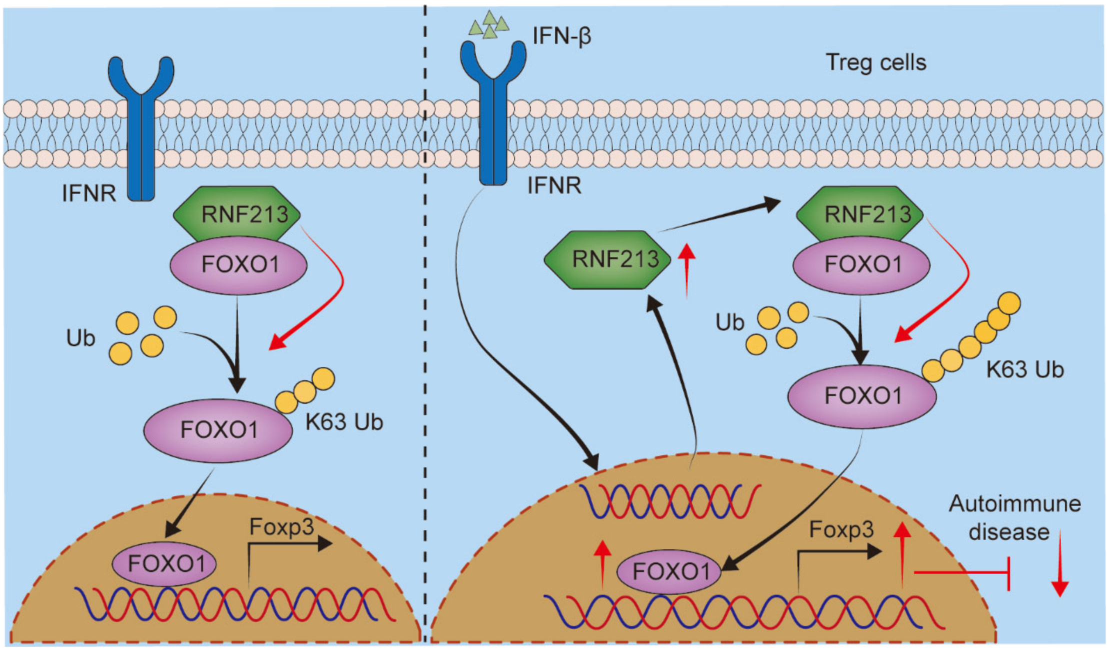

Supplementary figure 10. Illustration of a model on RNF213-mediated regulation of Treg cell differentiation. During Treg differentiation, RNF213 enhances the nuclear localization of FOXO1 to facilitate Foxp3 expression and promote Treg cell stability through the regulation of K63-linked polyubiquitination of FOXO1. Additionally, IFN-β augments Treg differentiation by upregulating RNF213 expression, thereby playing a therapeutic role in multiple sclerosis.

Supplementary Table 1. Antibodies

Antigen	Reactivity	Label	Clone	Manufacture	Use	Antibody dilutions
CD3	M	APC-eFluor 780	145-2C11	eBioscience	FCM	N/A
CD4	M	Percp-Cy5.5	RM4-5	eBioscience	FCM	N/A
CD8	M	APC	53-6.7	eBioscience	FCM	N/A
IFN-γ	M	eFluor 450	XMG1.2	eBioscience	FCM	N/A
IL-17A	M	FITC	eBio17B7	eBioscience	FCM	N/A
Foxp3	M	FITC	FJK-16s	eBioscience	FCM	N/A
CD25	M	PE	PC61.5	eBioscience	FCM	N/A
CD69	M	PE	H1.2F3	eBioscience	FCM	N/A
CD44	M	FITC	IM7	eBioscience	FCM	N/A
CD62L	M	PE	MEL-14	eBioscience	FCM	N/A
CD45.1	M	PE-Cy7	A20	eBioscience	FCM	N/A
CD45.2	M	APC	104	eBioscience	FCM	N/A
CD3	H	APC	OKT3	eBioscience	FCM	N/A
CD4	H	FITC	RPA-T4	eBioscience	FCM	N/A
IFN-γ	H	PE	4S.B3	eBioscience	FCM	N/A
IL-17A	H	PE-Cy7	eBio64DEC17	eBioscience	FCM	N/A
Foxp3	H	Percp-Cy5.5	PCH101	eBioscience	FCM	N/A
CD69	H	PE	FN50	eBioscience	FCM	N/A
CD44	H	FITC	IM7	eBioscience	FCM	N/A
CD62L	H	eFluor 450	DREG56	eBioscience	FCM	N/A
Ubiquitin	H/M		P4D1	CST	WB	1:1000
K63-linkage Specific Polyubiquitin	H/M		D7A11	CST	WB	1:1000
K48-linkage Specific Polyubiquitin			D9D5	CST	WB	1:1000
FLAG	H/M		D6W5B	CST	WB	1:2000
HA	H/M		C29F4	CST	WB	1:2000
Myc	H/M		9B11	CST	WB	1:2000
RNF213	H/M		cat. no. PA5-51902	Invitrogen	WB	1:500
FOXO1	H/M		C29H4	CST	WB	1:1000
p-FOXO1	H/M		cat. no. 9464	CST	WB	1:1000
Lamin B	H/M		E6M5T	CST	WB	1:2000

β-Actin	H/M		D6A8	CST	WB	1:2000

M, Mouse; H, Human

Supplementary Table 2. Gene-specific primers used for qRT-PCR

Gene	Forward primer	Reverse primer
hRnf213	GGAAAGGAAACCTCTGAACTCGG	CTCGTTCTGGTCTCTGAGCATG
hFoxp3	GGCACAATGTCTCCTCCAGAGA	CAGATGAAGCCTTGGTCAGTGC
hTgfb1	TACCTGAACCCGTGTTGCTCTC	GTTGCTGAGGTATCGCCAGGAA
hIl10	TCTCCGAGATGCCTTCAGCAGA	TCAGACAAGGCTTGGCAACCCA
hTgfb3	CTAAGCGGAATGAGCAGAGGATC	TCTCAACAGCCACTCACGCACA
hFoxo1	CTACGAGTGGATGGTCAAGAGC	CCAGTTCCTTCATTCTGCACACG
hActb	GTCTCCTCTGACTTCAACAGCG	ACCACCCTGTTGCTGTAGCCAA
mRnf213	TCACTGGAGCACCTACGGAAAC	TCTCAAACTCGCCCAGCACATTC
mFoxp3	CCTGGTTGTGAGAAGGTCTTCG	TGCTCCAGAGACTGCACCACTT
mTgfb1	TGATACGCCTGAGTGGCTGTCT	CACAAGAGCAGTGAGCGCTGAA
mIl10	CGGGAAGACAATAACTGCACCC	CGGTTAGCAGTATGTTGTCCAGC
mTgfb3	AAGCAGCGCTACATAGGTGGCA	GGCTGAAAGGTGTGACATGGAC
mFoxo1	CTACGAGTGGATGGTGAAGAGC	CCAGTTCCTTCATTCTGCACTCG
mActb	CATTGCTGACAGGATGCAGAAGG	TGCTGGAAGGTGGACAGTGAGG

M, Mouse; H, Human
