# Supplementary material for: RNF213 promotes Treg cell differentiation by facilitating K63-linked ubiquitination and nuclear translocation of FOXO1
Source: Nat Commun. 2024 Jul 16;15:5961. doi: 10.1038/s41467-024-50392-z (PMC11252262; doi:10.1038/s41467-024-50392-z)
Supplement: Supplementary file 3 — Reporting Summary [file 41467_2024_50392_MOESM3_ESM.pdf]

nature portfolio

Corresponding author(s): Shengfeng Hu, Xiaomin Li, Huili Zhang

Last updated by author(s): Jun 25, 2024

Reporting Summary

Nature Portfolio wishes to improve the reproducibility of the work that we publish. This form provides structure for consistency and transparency in reporting. For further information on Nature Portfolio policies, see our Editorial Policies and the Editorial Policy Checklist.

Statistics

For all statistical analyses, confirm that the following items are present in the figure legend, table legend, main text, or Methods section.

n/a	Confirmed	
☐	☒	The exact sample size (*n*) for each experimental group/condition, given as a discrete number and unit of measurement
☐	☒	A statement on whether measurements were taken from distinct samples or whether the same sample was measured repeatedly
☐	☒	The statistical test(s) used AND whether they are one- or two-sided<br>*Only common tests should be described solely by name; describe more complex techniques in the Methods section.*
☒	☐	A description of all covariates tested
☐	☒	A description of any assumptions or corrections, such as tests of normality and adjustment for multiple comparisons
☐	☒	A full description of the statistical parameters including central tendency (e.g. means) or other basic estimates (e.g. regression coefficient) AND variation (e.g. standard deviation) or associated estimates of uncertainty (e.g. confidence intervals)
☐	☒	For null hypothesis testing, the test statistic (e.g. *F*, *t*, *r*) with confidence intervals, effect sizes, degrees of freedom and *P* value noted<br>*Give P values as exact values whenever suitable.*
☒	☐	For Bayesian analysis, information on the choice of priors and Markov chain Monte Carlo settings
☒	☐	For hierarchical and complex designs, identification of the appropriate level for tests and full reporting of outcomes
☒	☐	Estimates of effect sizes (e.g. Cohen's *d*, Pearson's *r*), indicating how they were calculated

Our web collection on statistics for biologists contains articles on many of the points above.

Software and code

Policy information about availability of computer code

Data collection	Image Lab (BioRad, Version 5.1) for Western Blot; FACSDiva (BD, version 8.0.1) for FACS analysis; QuantStudio (Applied Biosystems, version 1.1) for quantitative Realtime PCR.
Data analysis	Flowjo (Window version, version 10.8.1); Graghpad Prism 8.0; Image lab (version 5.1); DESeq2 (version 1.20.0); GENE-E (version 3.0.215); GSEA (version 3.0), Perseus (Version 1.6.0.7).

For manuscripts utilizing custom algorithms or software that are central to the research but not yet described in published literature, software must be made available to editors and reviewers. We strongly encourage code deposition in a community repository (e.g. GitHub). See the Nature Portfolio guidelines for submitting code & software for further information.

Data

Policy information about availability of data

All manuscripts must include a data availability statement. This statement should provide the following information, where applicable:

- Accession codes, unique identifiers, or web links for publicly available datasets
- A description of any restrictions on data availability
- For clinical datasets or third party data, please ensure that the statement adheres to our policy

The RNA-seq data supporting the findings of this study have been deposited in the Gene Expression Omnibus at the National Center for Biotechnology Information and will be available under accession number GSE195541 and GSE66763; The mass spectra were processed and peptide identification was performed using the

Andromeda search engine found in Perseus (Version 1.6.0.7) against the UniProt mouse protein sequence database (UP000000589). The MS proteomics data have been deposited to the ProteomeXchange Consortium via the PRIDE partner repository with the dataset identifier PXD018701.

Research involving human participants, their data, or biological material

Policy information about studies with human participants or human data. See also policy information about sex, gender (identity/presentation), and sexual orientation and race, ethnicity and racism.

Reporting on sex and gender	In our study, MS patients between 10 and 65 years old were included. MS patients as IFN-β-responsive or non-responsive according to the previous description (Inoue, M. et al. Nat Neurosci 19, 1599-1609 (2016).). The sex/gender was not considered in our study design. But most of the patients we collect are women.
Reporting on race, ethnicity, or other socially relevant groupings	N/A
Population characteristics	The information for Age and Gender was not statistically significant between disease and control groups. The sex/gender was not considered in our study design. But most of the patients we collect are women.
Recruitment	The diagnosis of MS and disease severity was based on the The sex/gender was not considered in our study design. But most of the patients we collect are women.
Ethics oversight	Informed consent was obtained in accordance with the Declaration of Helsinki and the Institutional Review Board of the Southern Medical University. Written informed consents were obtained from all participants for the use of PBMC samples.

Note that full information on the approval of the study protocol must also be provided in the manuscript.

Field-specific reporting

Please select the one below that is the best fit for your research. If you are not sure, read the appropriate sections before making your selection.

☒ Life sciences ☐ Behavioural & social sciences ☐ Ecological, evolutionary & environmental sciences

For a reference copy of the document with all sections, see nature.com/documents/nr-reporting-summary-flat.pdf

Life sciences study design

All studies must disclose on these points even when the disclosure is negative.

Sample size	The sample sizes are determined based on the prevailing and widely accepted practice and similar designed experiments and results generated in the lab on the cellular immunology analyzes. No statistical method was used to pre-determine sample size. In addition, the sample sizes for each experiment have been detailed in the figure legends and methods.
Data exclusions	No valid data were excluded.
Replication	All experiments are reliably reproduced. All data displayed included at minimum two independent experiments, and minimum three biological replicates for each independent experiment.
Randomization	Aged and sex matched mice with desired genotype were chosen randomly. For example, In all in vivo experiments, different group of mice are mixed in one cage, and treated equally without knowing or identifying the identity of the mice. The mice were then identified upon the completion of the experiments.
Blinding	All samples were labeled with simple numbers. The person who performed the experiments did not know what the samples were until after data were collected and analyzed.

Reporting for specific materials, systems and methods

We require information from authors about some types of materials, experimental systems and methods used in many studies. Here, indicate whether each material, system or method listed is relevant to your study. If you are not sure if a list item applies to your research, read the appropriate section before selecting a response.

Materials & experimental systems

n/a	Involved in the study	
☐	☒	Antibodies
☐	☒	Eukaryotic cell lines
☒	☐	Palaeontology and archaeology
☐	☒	Animals and other organisms
☒	☐	Clinical data
☒	☐	Dual use research of concern
☒	☐	Plants

Methods

n/a	Involved in the study	
☒	☐	ChIP-seq
☐	☒	Flow cytometry
☒	☐	MRI-based neuroimaging

Antibodies

Antibodies used

List of antibodies used in the Method section (with Antigen, Reactivity Label, Clone, Manufacture, Use, as applicable)
CD3, M, APC-eFluor 780, 145-2C11, eBioscience, FCM;
CD4, M, Percp-Cy5.5, RM4-5, eBioscience, FCM;
CD8, M, APC, 53-6.7, eBioscience, FCM;
IFN-γ, M, eFluor 450, XMG1.2, eBioscience, FCM;
IL-17A, M, FITC, eBio17B7, eBioscience, FCM;
Foxp3, M, FITC, FJK-16s, eBioscience, FCM;
CD25, M, PE, PC61.5, eBioscience, FCM;
CD69, M, PE, H1.2F3, eBioscience, FCM;
CD44, M, FITC, IM7, eBioscience, FCM;
CD62L, M, PE, MEL-14, eBioscience, FCM;
CD45.1, M, PE-Cy7, A20, eBioscience, FCM;
CD45.2, M, APC, 104, eBioscience, FCM;
CD3, H, APC, OKT3, eBioscience, FCM;
CD4, H, FITC, RPA-T4, eBioscience, FCM;
IFN-γ, H, PE, 4S.B3, eBioscience, FCM;
IL-17A, H, PE-Cy7, eBio64DEC17, eBioscience FCM;
Foxp3, H, Percp-Cy5.5, PCH101, eBioscience, FCM;
CD69, H, PE, FN50, eBioscience, FCM;
CD44, H, FITC, IM7, eBioscience, FCM;
CD62L, H, eFluor 450, DREG56, eBioscience, FCM;
Ubiquitin, H/M, P4D1, CST, WB;
K63-linkage Specific Polyubiquitin, H/M, D7A11, CST, WB;
K48-linkage Specific Polyubiquitin, D9D5, CST, WB;
FLAG, H/M, D6W5B, CST, WB;
HA, H/M, C29F4, CST, WB;
Myc, H/M, 9B11, CST, WB;
RNF213, H/M, cat. no. PA5-51902, Invitrogen, WB;
FOXO1, H/M, C29H4, CST, WB;
p-FOXO1, H/M, cat. no. 9464, CST, WB;
Lamin B, H/M, E6M5T, CST, WB;
β-Actin, H/M, D6A8, CST, WB.

Validation

All the antibodies are validated by the manufacturers or previously done by others and our laboratory. Whenever it is possible, we have validated them with established methods and reagents including gene knockout cells. These antibodies were used in flow cytometry and western blot/IP experiments to detect the endogenous protein level of ex vivo primary mouse cells.

Eukaryotic cell lines

Policy information about cell lines and Sex and Gender in Research

Cell line source(s)

The 293T cell line was purchased from ATCC: 293T (ATCC® CRL-3216TM).

Authentication

The 293T cell line was not authenticated.

Mycoplasma contamination

The 293T cell line was not tested for mycoplasma contamination.

Commonly misidentified lines
(See ICLAC register)

No commonly misidentified cell lines were used.

Animals and other research organisms

Policy information about studies involving animals; ARRIVE guidelines recommended for reporting animal research, and Sex and Gender in Research

Laboratory animals

C57BL/6 mice (Wild type, WT) were from the Lab Animal Center of Southern Medicine University (Guangzhou, China). RNF213-

Laboratory animals	deficient (Rnf213-/-, Cat. NO. S-KO-12190), Rnf213flox/flox (Rnf213fl/fl, Cat. NO. S-CKO-13591) and Foxo1fl/fl (S-CKO-12115) mice on the C57BL/6J background were generated by Cyagen Biosciences Inc. (Guangzhou, China) using CRISPR-Pro technology. CD4-Cre (CD4Cre, Cat. NO. SJ-022071) and Foxp3Cre (Cat. NO. SJ-004337), CD45.1+ (Cat. NO. KI-210226) and Rag1-/- (Cat. NO. KI-00069) mice were purchased from the Shanghai Research Center for Model Organisms (Shanghai, China). Rnf213fl/fl mice were crossed with CD4Cre or Foxp3Cre mice to generate Rnf213fl/flCD4Cre or Rnf213fl/flFoxp3Cre mice. Foxo1fl/fl mice were crossed with Foxp3Cre mice to generate Foxo1fl/flFoxp3Cre mice. Rnf213fl/flFoxp3Cre mice were crossed with Foxo1fl/flFoxp3Cre to generate Rnf213fl/flFoxo1fl/flFoxp3Cre. RNF213-transgenic (Rnf213Tg) mice were generated by Biocytogen (China). In brief, the pBS31- RNF213-Flag plasmid was co-electroporated with a recombinase expression vector into ES cells that were expressing the M2rtTA tetracycline-responsive transactivator under control of the ROSA26 promoter. Transgene expression was induced by feeding the mice 2 mg/ml doxycycline in their drinking water supplemented with 10 mg/ml sucrose. To generate mice with Treg-cell-specific overexpression of RNF213-Flag (Rnf213TgFoxp3Cre mice), Rnf213Tg mice were crossed with Foxp3-Cre mice. All mice were all C57BL/6 background and maintained in the Lab Animal Center of Southern Medicine University under specific pathogen-free (SPF) conditions. Animals were in a 12-h light/dark cycle beginning at 7am (light) and 7pm (dark). Ambient temperature was 20–22°C with humidity 40–60%. Mice were euthanized using CO_2 asphyxiation dispensed from a fixed pressure regulator and inline restrictor controlling gas flow. CO_2 flow was maintained for at least 5min. Death was verified following euthanasia by monitoring cessation of heartbeat and respiration, as well as a toe pinch reflex. All mice were used at an age of 6–12 weeks and were randomly divided into different groups. Female mice were used for all experiments, unless otherwise stated.
Wild animals	No wild animal were used in the study.
Reporting on sex	No field samples were used in the study.
Field-collected samples	No field samples were used in the study.
Ethics oversight	All animal experiments were conducted in accordance with protocols approved by the Medical Ethics Board and the Biosafety Management Committee of Southern Medical University.

Note that full information on the approval of the study protocol must also be provided in the manuscript.

Plants

Seed stocks	N/A
Novel plant genotypes	N/A
Authentication	N/A

Flow Cytometry

Plots

Confirm that:

☒ The axis labels state the marker and fluorochrome used (e.g. CD4-FITC).

☒ The axis scales are clearly visible. Include numbers along axes only for bottom left plot of group (a 'group' is an analysis of identical markers).

☒ All plots are contour plots with outliers or pseudocolor plots.

☒ A numerical value for number of cells or percentage (with statistics) is provided.

Methodology

Sample preparation	For intracellular cytokine staining assays, T cells isolated from spleen or nervous system of mice, or from in vitro cultures were stimulated for 1.5 hours with 100 mg/ml MOG(35-55) or PMA (50 ng/ml, Thermo Fisher Scientific, USA) and ionomycin (500 ng/ml, Thermo Fisher Scientific), before Brefeldin A (10 µg/ml, eBioscience, USA) was added to the culture for 3.5 hours more. As previously described48, for surface staining, cells were harvested, washed, and stained for 30 min on ice with mixtures of fluorescently conjugated mAbs or isotype-matched controls. For intracellular cytokine staining (ICS), cells were stained for surface molecules, fixed 20 min in IC Fixation buffer (Thermo Fisher Scientific), and incubated for 1 h in permeabilization buffer (Thermo Fisher Scientific) with appropriate mAbs of mice.
Instrument	Cell phenotype was analyzed by flow cytometry on a flow cytometer (BD LSR II) (BD Biosciences, USA) or Attune NxT (Thermo Fisher Scientific).
Software	Data were acquired as the fraction of labeled cells within a live-cell gate and analyzed using FlowJo software (Tree Star).

Cell population abundance	The numbers of sorted cell populations are typically more than 1 million with a purify of 95% or above, validated by flowcytometry analysis using relevant antibody staining.
Gating strategy	Preliminary FSC/SSC gating was used to gate on lymphocytes population and single cells. Specific T cell populations were gated based on the specific antibody staining as described in each experiment.

☒ Tick this box to confirm that a figure exemplifying the gating strategy is provided in the Supplementary Information.
